# Expression of senescence-related CD161 promotes extranodal NK/T cell lymphoma by affecting T cell phenotype and cell cycle

**DOI:** 10.1186/s10020-024-00969-7

**Published:** 2024-11-23

**Authors:** Chengxun Jin, Xin Li, Chaohe Zhang

**Affiliations:** 1 Department of Otolaryngology, The Second Hospital of Jilin University, No.4026, Yatai street, Nanguan District, Changchun, 130000 China; 2 Department of Radiology, The Second Hospital of Jilin University, Changchun, 130000 China; 3 Department of Tumor Hematology, The Second Hospital of Jilin University, No.4026, Yatai street, Nanguan District, Changchun, 130000 China

**Keywords:** Extranodal NK/T-cell lymphoma (ENKTL), Cell cycle, T cell phenotype, CD161, Senescence

## Abstract

**Purpose:**

The intention of this work is to probe the role of senescence-related gene CD161 in extranodal NK/T cell lymphoma (ENKTL).

**Methods:**

This study used H_2_O_2_ to establish three distinct in vitro oxidative stress aging models (NKL, SNT-8, and YT). Western blotting was employed to assess the levels of two iconic aging proteins, MMP1 and P53, and flow cytometry was utilized to investigate cell cycle and the expressions of CD4, CD8, and CD161. Cell viability was evaluated via the CCK-8 assay. The transcriptome analysis assessed the differential gene expression between the control and aging group of NKL. In vivo, we established a BALB/c mice aging tumor model. After 15 days, the mice were euthanized to harvest tumors. ELISA was employed to measure aging indicators in the mouse tissues. Flow cytometry was utilized to assess the levels of CD4, CD8, and CD161 in tumor samples. Hematoxylin-eosin (HE) staining was performed to evaluate the structure and cellular morphology of the tumor tissue.

**Results:**

In the NKL, SNT-8 and YT aging models, the levels of MMP1 and P53 proteins were significantly increased. Flow cytometry results indicated that all three cell types exhibited marked arrest in the G1 phase. Compared with the control group, the expressions of CD4 and CD161 in the aging group were significantly increased, while the expression of CD8 was decreased. Transcriptome analysis revealed 2,843 differentially expressed genes (DEGs) between the control and aging groups, with 2,060 up-regulated and 783 down-regulated genes identified. Following CD161 knockdown, cell viability of three cell types in the aging group was significantly reduced compared to the control group. The G1 phase of the cells was significantly interrupted. The expressions of CD4 and CD161 were significantly increased, and the expression of CD8 was decreased. However, in the aging + si-CD161 group, a partial alleviation of oxidative stress was observed with a reduction in CD161 expression levels. Animal experiments demonstrated that knockout of CD161 can inhibit tumor progression and partially mitigate oxidative stress.

**Conclusions:**

CD161 may inhibit ENKTL tumor development by regulating cell cycle and T-cell phenotype.

**Supplementary Information:**

The online version contains supplementary material available at 10.1186/s10020-024-00969-7.

## Introduction

Extranodal NK/T-cell lymphoma (ENKTL), also known as angiocentric T-cell lymphoma, is a rare extranodal non-Hodgkin lymphoma (NHL) that usually occurs in the upper respiratory tract. The WHO classification has selected the label “nasal type” (ENKTL-NT) for ENKTL because of its common presentation in the nasopharynx. Moreover, ENKTL-NT frequently spreads locally to the orbit, lymph nodes, and paranasal sinuses. In rare circumstances, it may even develop outside the nasopharynx. About 40% of patients have an overall survival of only 5 years. Roughly 1% of all NHL types and 10% of peripheral blood T-cell lymphomas are caused by ENKTL occurrence. Asia, Central America, and South America have higher rates of ENKTL (Ma et al. 2013; William and Armitage 2013; Cai et al. 2014; Haverkos et al. 2016), while China has higher rates of ENKTL than Western nations (Au et al. 2009; Yang et al. 2015; Vargo et al. 2017). In fact, despite pegaspargase being used to achieve certain remission in ENKTL, there has been no significant progress in the etiology and treatment of ENKTL in recent years. Moreover, the prognosis of ENKTL patients is poor, and up to 50% of patients will relapse. Although intensive combination chemotherapy has been implemented for ENKTL, the survival rate of patients with advanced ENKTL is still less than 30%. This highlights the urgent need to develop new treatments for ENKTL or explore its new mechanism of action (Brammer et al. 2018; Mel et al. 2019; Mundy-Bosse et al. 2022; Liang et al. 2023).

With the improvement of living conditions and medical innovation, people’s life span has been improved. Although the risk of cancer increases in patients over age 60, cancer rates actually decline by age 85. The reasons are not fully understood but may be related to a decrease in cell proliferation potential. Senescent cell buildup is a significant risk factor for a number of age-related illnesses, such as cancer (Harding et al. 2008; Childs et al. 2015; Campisi et al. 2019; DeSantis et al. 2019; Wang et al. 2020a, b; Maggiorani and Beausejour 2021; Siegel et al. 2021). Cellular senescence is one of the key processes of aging and is the link between aging and cancer. It is considered an effective means of preventing the malignant behavior of cancer cells. Because cellular senescence is the process in which cells stop dividing and lose their ability to proliferate. Therefore, cellular senescence can play a role in suppressing tumors to a certain extent. Senescent cells can secrete SASP factors, which further enhance the tumor suppressor effect by recruiting immune cells to promote immune clearance (Krizhanovsky et al. 2008; Campisi 2013; Perez-Mancera et al. 2014).

The prognostic factors of elderly ENKTL patients are worse than those of younger patients (Kim et al. 2016). Age is a continuous variable that affects the survival and treatment options of ENKTL patients (Liu et al. 2019). CD161 is a C-type lectin receptor expressed by NK and T cells. The expression of the senescence-related gene CD161 and its changes in CD8 + T cells may lead to abnormal immune function in the elderly (Yokoyama and Seaman 1993; Lanier et al. 1994; Battistini et al. 1997; Geest et al. 2018). Aging can affect the frequency and phenotype of NK cells, and age-induced changes in CD161 expression in NK cell subsets are more significant (Lopez-Sejas et al. 2016). Additionally, research has demonstrated that age has an impact on CD161 expression, which is also recognized for its significant role in various cancer types. At present, CD161 has been recognized as a prognostic biomarker for breast cancer (Weng et al. 2022). Moreover, the expression of CD161 has been demonstrated to have a significant correlation with the pathological and molecular characteristics of gliomas (Di et al. 2022; Mathewson et al. 2021a, b).However, it is unclear how CD161 works in ENKTL (Formentini et al. 2021). Based on this, this article studied the impact of CD161 on ENKTL and explored the role of CD161 in ENKTL by constructing an in vitro tumor cell aging model and an in vivo BALB/c mice aging tumor model, as well as transcriptome sequencing technology. It is anticipated that CD161 will exert a significant promoting effect in ENKTL; consequently, we propose that targeting CD161 may offer a novel therapeutic strategy for the treatment of this malignancy.

## Materials and methods

### Animals

A total of 24 male Balb/c mice (6–8 weeks, 20 ± 2 g) were acquired from Jilin University’s Animal Center. Every mouse was housed in a specific pathogen-free (SPF) animal laboratory, which maintained a 12-hour light/dark cycle and provided adequate food and water.

### Cell culture

NKL, SNT-8, and YT cell lines were purchased from Shanghai Hongshun Biological Co., Ltd. (Shanghai, China), Wuhan Pronoser Life Technology Co., Ltd. (Wuhan, China), and Guangzhou Aidi Gene Technology Co., Ltd. (Guangzhou, China), respectively. Cells were cultured in RPMI 1640 medium, supplemented with 10% fetal bovine serum and 1% penicillin/streptomycin. All cells were maintained in a humidified incubator at 37 °C with 5% CO_2_.

### Cell model construction and cell viability assay

Three distinct types of cells, demonstrating optimal morphology and growth characteristics, were collected and inoculated into 96-well plates at an appropriate cell density, with 3 duplicate wells for each type. Subsequently, the cells in 96-well plates were cultured in an incubator maintained at a constant temperature of 37 °C with an atmosphere of 5% CO_2_. H_2_O_2_ was administered to the cells at varying concentrations (0, 100, 200, and 400 µM/L) for different durations (0, 2, 4, and 8 h) to construct cellular oxidative stress aging models. Following treatment, the supernatant was removed and newly prepared CCK-8 solution was added. The mixture was then incubated in an incubator for 1 h. Absorbance readings were obtained at 450 nm using a microplate reader. Three independent experimental replicates were conducted.

### Flow cytometry analysis

The cell cycle was evaluated using flow cytometry. Initially, logarithmic growth phase cells were inoculated in 1 mL of medium at 1 × 10^6^ cells/mL in a 24-well plate. After the culture period, the cells were harvested and washed twice with cold PBS, followed by the fixation in cold 75% ethanol at 4 °C for over 4 h. Subsequently, the cell suspension was centrifuged at 1500 rpm for 5 min. The supernatant was carefully removed, and the pellet was treated with 400 µL of CCAA solution (PI staining solution, 50 µg/mL; add 100 µL RNase A, concentration 100 µg/mL). The mixture was incubated in the dark at 4 °C for 30 min and then detected employing a flow cytometer.

To assess protein expression via flow cytometry, the cell suspension was initially centrifuged and resuspended in PBS. Subsequently, target antibodies for detection were added and incubated at room temperature in a dark environment. Following a 4-fold dilution, cells were washed with PBS three times and centrifuged for 5 min, then resuspended in 200 µL PBS and detected using a flow cytometer. Data were analyzed using Flow Jo software.

### Cell transfection

Transfection was performed according to the instructions of Lipofectamine™2000. Specifically, cells were cultured overnight, and transfection was conducted when the cells reached 70% confluence. Subsequently, two mixtures were prepared and allowed to stand for 5 min. The first mixture comprised 1 µL of Lipofectamine 2000 and 100 µL of serum-free RPMI 1640, while the second mixture contained 100 pM of siRNAs dissolved in 100 µL of serum-free RPMI 1640. After preparation, the two mixtures were mixed and incubated at room temperature for 20 min. The aforementioned mixture was added to the wells containing cells and incubated at 37 °C with 5% CO_2_ for 6 h. Following a 6-hour transfection period, the medium was replaced with a complete medium supplemented with 10% FBS and 1% penicillin/streptomycin, after which the transfected cells were cultured overnight for subsequent experiments. QRT-PCR was employed to assess the silencing efficiency of the following groups: Control, si-NC, si-CD161-1, si-CD161-2, and si-CD161-3. The sequences of the siRNAs are presented in Table 1.

**Table 1:** The primers of siRNA and mRNA

	Sense(5’-3’)	Antisense(5’-3’)
mCD4	GTGTCTACTGAGTGAAGGTGATAAGG	GCCCAAGGAAACCCAGAAAGC
hCD4	TTTCATTGGGCTAGGCATCTTCTTC	AGGACACTGGCAGGTCTTCTTC
mCD8	GACTTCGCCTGTGATATCTACATCTG	CGTCTTCGGTTCCTGTGGTTG
hCD8	GCCAGGAACAAGCAGTGAGAAC	TACAAGGAGCACGAGGCAGAC
mCD161	GACCAAGAAGAACTGAGATTCCTACTG	ATGTCTGGCAATGTGAACCTTAGTC
hCD161	AAGTTCTTCACCTTCATCTCTTCCTC	TCAACCCAGTAACAACCAAGACAAG
siCD161-1	AGAGAAAUGCUUGUUAUUUTT	AAAUAACAAGCAUUUCUCUTT
siCD161-2	AGAUAAGGAUGAAUUGAUATT	UAUCAAUUCAUCCUUAUCUTT
siCD161-3	CAUUCAACAGAGCAGGAAUTT	AUUCCUGCUCUGUUGAAUGTT

### qRT-PCR

After collecting the transfected cells, Trizol reagent was added to extract the total cellular RNA. Reverse transcription was conducted to synthesize cDNA under the following conditions: 25 °C for 5 min, 50 °C for 15 min, and 85 °C for 5 min, followed by storage at 4 °C. Subsequently, qRT-PCR was conducted utilizing ChamQ Universal SYBR qPCR master Mix under the following conditions: pre-denaturation at 95 °C for 30 s, followed by 40 cycles of denaturation at 95 °C for 10 s, and annealing/extension at 60 °C for 30 s. GAPDH served as an internal reference for quantifying mRNA levels in each sample. The primer sequences for qRT-PCR are presented in Table 1.

### Western blotting

The cells were lysed using RIPA lysis buffer. Following centrifugation of the lysate, total cellular protein was extracted. A BCA protein quantitative detection kit was utilized to determine the concentration of total protein. The protein samples were then mixed with loading buffer in proportion and denatured by heating. Subsequently, quantified denatured proteins were added to the pre-prepared SDS-PAGE gel to perform electrophoresis. Subsequent to electrophoresis, the protein samples were transferred onto a PVDF membrane, followed by blocking with 5% skim milk for 1 h. The membrane was promptly washed with TBST three times (10 min each), followed by incubation with specific primary antibodies (GAPDH: ABclonal, A19056, 1:50000; MMP1: ABclonal, A22080, 1:5000; P53: ABclonal, A25915, 1:1000) on a shaker at 4 °C overnight. The following day, the membrane was incubated with a secondary antibody (1:5000 dilution) for 1 h, followed by three washes with TBST buffer. The protein blots on the membranes were visualized using ECL chemiluminescence detection kits in a gel imaging system. Image J software was employed to analyze and compare the expression levels of proteins.

### Transcriptome sequencing

Initially, the NKL cell oxidative stress aging model was constructed via H_2_O_2_ stimulation (200 µM/L, 8 h). The total RNA of the cell model was extracted and the concentration and purity of extracted RNA were determined using Nanodrop 2000. RNA integrity was evaluated through agarose gel electrophoresis, while the RIN value was calculated using an Agilent 2100 system. MRNA was isolated from total RNA via Oligo (dT) magnetic beads and poly (A) for A-T base pairing. Subsequently, mRNA fragments were generated to synthesize single-stranded cDNA. After purification and recovery, sticky end repair was conducted. The “A” base was appended to the 3’ end of the cDNA for fragment sorting purposes. Following quality control measures, high-throughput sequencing was executed on an Illumina platform.

### Animal experiments

A total of 24 mice were randomly separated into the following groups: si-NC group (*n* = 6), si-CD161 group (*n* = 6), Aging + si-NC group (*n* = 6), and Aging + si-CD161 group (*n* = 6). The aging model was established via intraperitoneal administration of D-galactose (0.1 mL/10 g), with injections performed daily for a period of 8 weeks. The tumor mouse model was established by subcutaneously injecting NKL cell suspensions to facilitate tumor formation in BALB/c mice. Throughout the experiment, tumor progression in each group of mice was monitored. Fifteen days post-treatment, the mice were anesthetized via intraperitoneal injection of ketamine (75 mg/kg) and xylazine (8 mg/kg), followed by euthanasia through cervical dislocation to obtain tumors.

### HE staining

After the acquisition of subcutaneous tumors from mice, tumor tissues were fixed and dehydrated using 4% paraformaldehyde. Paraffin blocks of tumor tissue were prepared, followed by deparaffinization and rehydration of the tissue sections. The tumor tissues were subsequently stained with hematoxylin-eosin. Finally, xylene was employed as a transparent agent to replace the dehydrating agent, and the sections were sealed with neutral resin for microscopic observation of their morphological structure.

### Statistical analysis

Gene quantitative analysis was performed using RSEM software. DESeq2 software was used for gene difference analysis, and the screening threshold was: |log2FC|≥1& *p* adjust < 0.05. Data analysis was performed using GraphPad Prism 8 and Microsoft Excel 2016 software. For normally distributed data, an unpaired t-test was utilized for statistical comparisons; conversely, a non-parametric Mann-Whitney test was applied when normality assumptions were not met. Data are presented as mean ± SEM (n = number of experiments) unless otherwise specified. Significance levels were defined as *p < 0.05, **p < 0.01, ***p < 0.001, and ****p < 0.0001.

## Results

### Construction and validation analysis of in vitro cell models

**Fig. 1:**
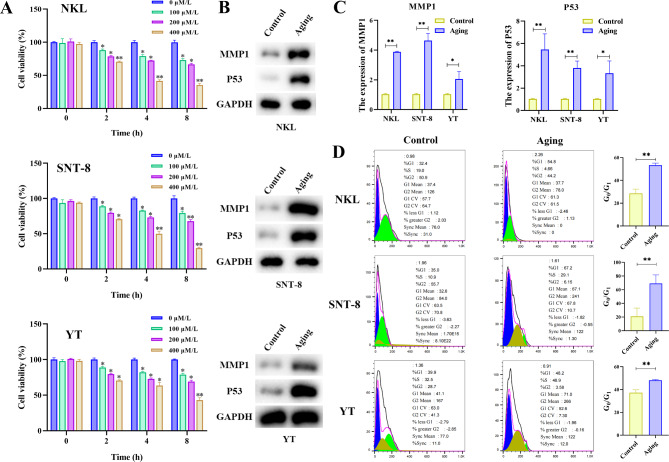
(**A**) CCK-8 method detects the cell viability of three types of cells (NKL, SNT-8, and YT) stimulated by different concentrations of H_2_O_2_ for different times. (**B**) Western blotting detects the expression of aging marker proteins and related statistical data in three different cells (NKL, SNT-8, and YT, the same below) and different groups (Control group and Aging group, the same below). (**C**) The protein expression levels of MMP1 and p53 in three different cells and different groups. (**D**) Flow cytometry analyzes the cell cycle and related statistical data of different groups of three cells. *p < 0.05, **p < 0.01, compared with the Control group (*n* = 3/per group)

First, different concentrations of hydrogen peroxide (0, 100, 200, 400 µM/L) were administered to treat NKL, SNT-8, and YT cells for 4 different time durations (0, 2, 4, 8 h) to construct a cellular oxidative stress aging model. The cell viability was then determined via the CCK-8 method (Fig. 1A).

**Fig. 2:**
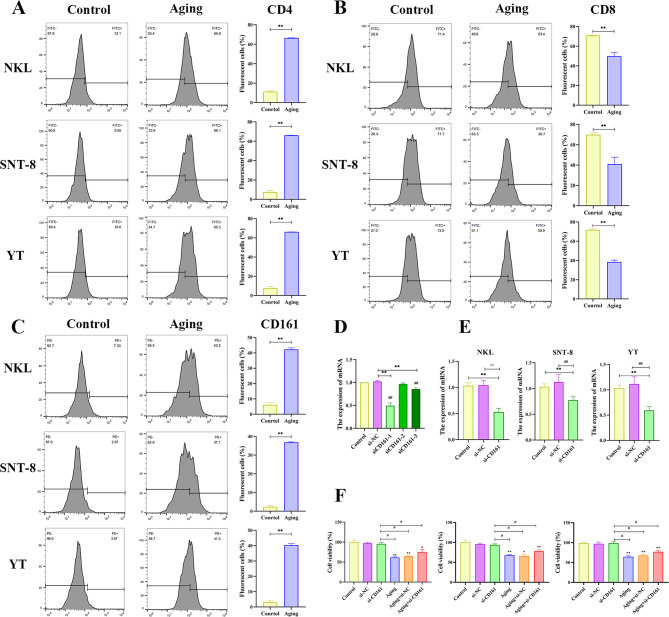
(**A-C**) Flow cytometry was used to analyze the expression of CD4, CD8, and CD161 and related statistical data of three types of cells (NKL, SNT-8, and YT) in different groups (Control group and Aging group). (**D**) Verification of transfection efficiency of three different sequences of si-CD161. (**E**) Validation analysis of transfection efficiency of si-CD161 in three different cells. (**F**) CCK-8 method detects the viability of the three types of cells (NKL, SNT-8, and YT) in different groups (Control group, si-NC group, si-CD161 group, Aging group, Aging + si-NC group, and Aging + si-CD161 group). *p < 0.05, **p < 0.01, compared with the Control group, #*p* < 0.05, ##*p* < 0.01, compared with the si-CD161 group (*n* = 3/per group)

The results showed that at 200 µM/L, cell viability decreased significantly at 8 h. It reached a damaged state but also had a certain degree of cell viability, which was in line with the experimental research conditions, so this condition was selected for subsequent experiments. Tumor Protein 53 (TP53) is a pivotal tumor suppressor gene, and it is widely acknowledged that elevated levels of its encoded protein p53 can significantly enhance cellular senescence. Moreover, Matrix Metalloproteinase 1 (MMP1) has been demonstrated to have a significant correlation with cellular aging across various cell types and exhibits elevated expression levels in numerous tumor tissues. Therefore, Western blotting was employed to assess the expression levels of these two hallmark proteins associated with cellular senescence. The findings demonstrated the aging group exhibited considerably higher levels of p53 and MMP1 protein expression (Fig. 1B,C). In comparison to the control group, flow cytometry analysis of cell cycle distribution revealed an increase in the G1 phase within the aging group. DNA replication in cells from the aging group was arrested at the G1 phase, leading to inhibited proliferation of all three tumor cell lines (Fig. 1D). Additionally, the flow cytometry detection results demonstrated that the proportions of CD161 and CD4 in the three cell aging groups were substantially higher than in the Control group, whereas the proportion of CD8 was expressively lower (Fig. 2A-C).

**Fig. 3:**
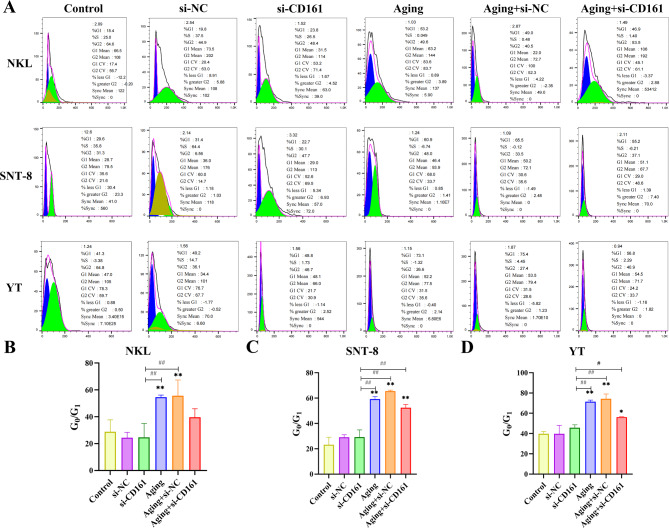
(**A**) Flow cytometry was used to analyze the cell cycles of the three different groups of cells (NKL, SNT-8, and YT) (Control group, si-NC group, si-CD161 group, Aging group, Aging + si-NC group, and Aging + si-CD161 group). Cell cycle statistical results of different groups (Control group, si-NC group, si-CD161 group, Aging group, Aging + si-NC group, and Aging + si-CD161 group) of NKL cells (**B**) SNT-8 cells (**C**) and YT cells (**D**). *p < 0.05, **p < 0.01, compared with the Control group, #*p* < 0.05, ##*p* < 0.01, compared with the si-CD161 group (*n* = 3/per group)

**Fig. 4:**
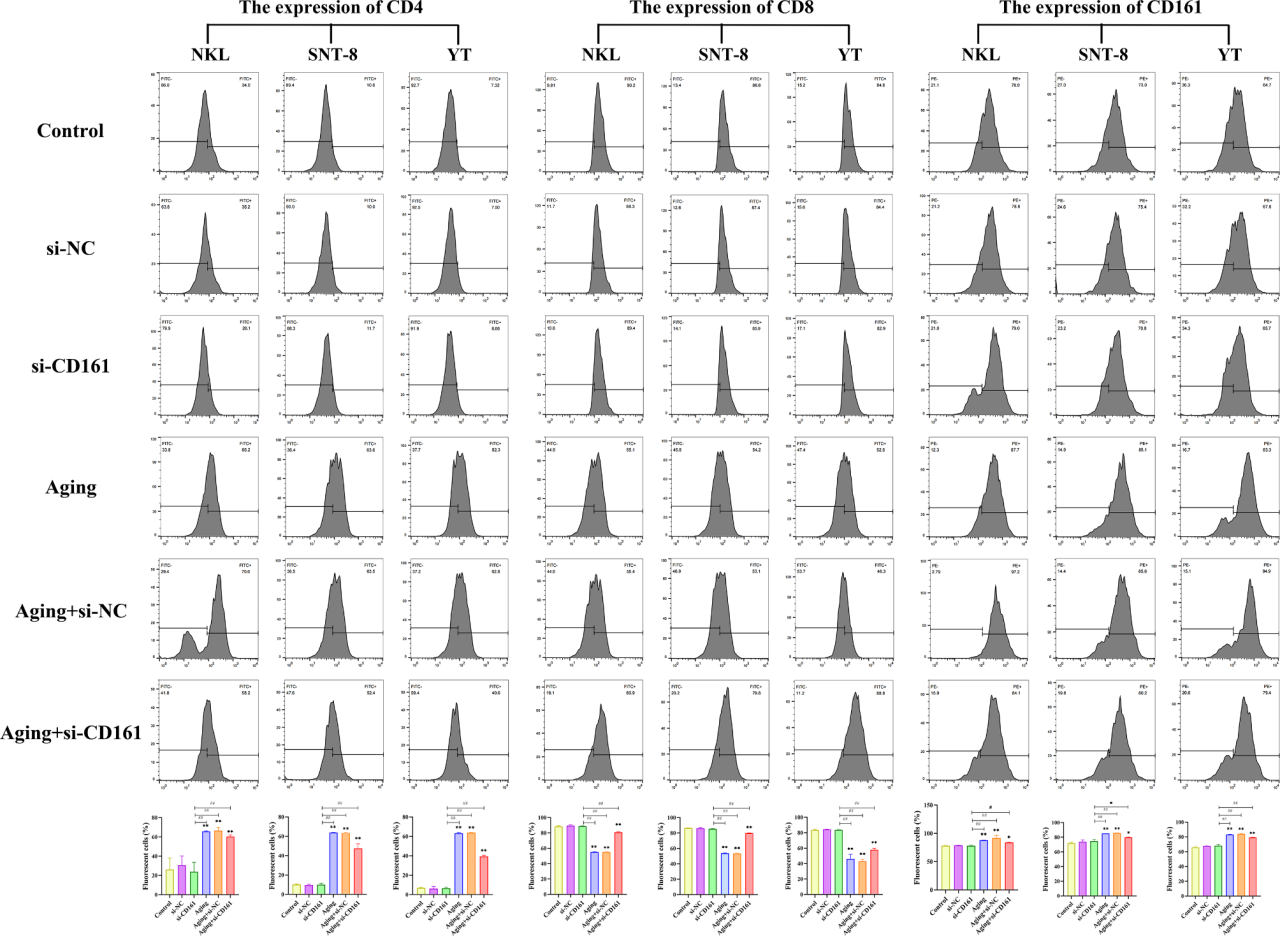
The expression of CD4, CD8 and CD161 in three kinds of cells (NKL, SNT-8, and YT) in different groups (including Control group, si-NC group, si-CD161 group, Aging group, Aging + si-NC group, Aging + si-CD161 group) and related statistical data of each group. *p < 0.05, **p < 0.01, compared with the Control group, #*p* < 0.05, ##*p* < 0.01, compared with the si-CD161 group (*n* = 3/per group)

CD161 small interfering RNA (si-CD161) was synthesized, and mRNA was extracted from NKL cells transfected with three distinct siRNAs (siCD161-1, siCD161-2, siCD161-3), followed by the assessment of silencing efficiency using qRT-PCR. It was found that the silencing efficiency of transfected siCD161-1 was the highest, so siCD161-1 was selected for subsequent experiments (Fig. 2D), and the mRNA expression levels of the three cells after silencing CD161 were detected (Fig. 2E).The three cell types (NKL, SNT-8, and YT) were categorized into six groups, encompassing the Control group, si-NC group, si-CD161 group, Aging group, Aging + si-NC group, and Aging + si-CD161 group. The CCK-8 method was employed to assess cell viability. Similar experimental results were obtained for the three types of cells. Compared to the Control group, there was no significant difference between the si-NC group and the si-CD161 group, while the cell viability of the Aging + si-CD161 group, Aging group, and Aging + si-NC group was significantly reduced. Compared with the si-CD161 group, the cell viability of the Aging group, Aging + si-NC group, and Aging + si-CD161 group decreased (Fig. 2F). These findings indicated that knocking down CD161 significantly inhibited cell proliferation and suggested an anti-apoptotic role for CD161 in the aging models of NKL, SNT-8, and YT cells. To elucidate the potential mechanisms underlying the inhibition of cell proliferation, flow cytometry was employed to analyze the cell cycle across all groups. The results revealed a significant increase in the proportion of G1 phase cells in each group, indicating that cells were arrested at this phase, thereby inhibiting further proliferation. Compared to the Control group, no significant differences were observed between the si-NC and si-CD161 groups. Cell viability was diminished in the Aging + si-CD161 group as well as in both aging-related conditions (Aging and Aging + si-NC), reflecting inhibited proliferation overall. In comparison to the si-CD161 group, a marked reduction in cell viability was noted within the Aging + si-CD161 cohort. (Fig. 3). It suggested that CD161 is closely related to aging, and knocking down CD161 can significantly reduce the survival rate of NKL, SNT-8, and YT cells. The expression of associated proteins was then determined by flow cytometry, and the outcomes demonstrated a correspondence with the cell cycle results (Fig. 4). Compared with the Control group, there was no significant difference in the expression of CD4, CD8, and CD161 in the si-NC group and si-CD161 group. The expression of CD161 and CD4 of the three kinds of cells in the Aging + si-CD161 group, Aging group, and Aging + si-NC group were much higher, whereas CD8 was noticeably lower.

### In vivo verification through animal experiments

**Fig. 5:**
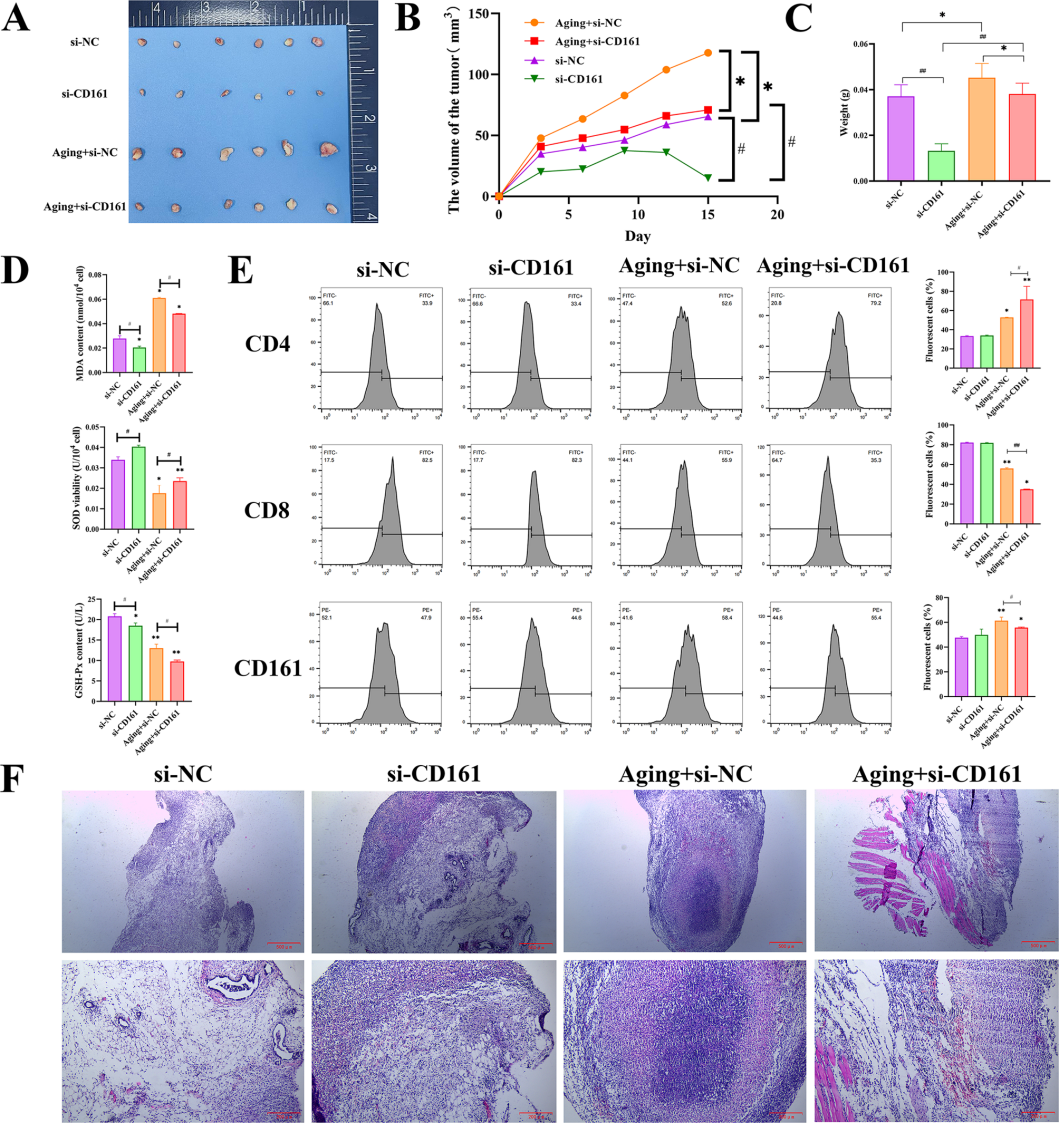
(**A**) Photos of mice tumors obtained from different groups (si-NC group, si-CD161 group, Aging + si-NC group, and Aging + si-CD161 group. (**B**) Line chart of tumor volume changes over time in mice in different groups. (**C**) Statistical chart comparing tumor weight of mice in different groups. (**D**) The ELISA method was used to detect the MDA content, SOD activity, and GSH-Px content in the serum of mice in different groups. (**E**) Flow cytometry analysis of CD4, CD8, and CD161 expression and related statistical data in tumor tissues of mice in different groups. (**F**) HE staining images of tumor tissues of mice in different groups. *p < 0.05, **p < 0.01, compared with the Control group, #*p* < 0.05, ##*p* < 0.01, compared with the si-CD161 group (*n* = 3/per group)

BALB/c mice aging model was conducted via continuous intraperitoneal injection of β-galactose for 8 weeks, and BALB/c mice tumor model was conducted via subcutaneous injection of NKL tumor cells. The experiment was divided into 4 groups: si-NC group; si-CD161 group; Aging + si-NC group; Aging + si-CD161 group. Tumor size was recorded every three days, and the obtained tumor photos of mice in every group are shown in Fig. 5A. Experimental results showed that after injecting tumor cells into mice, the tumor size of mice in each group continued to increase as time went on, except for the si-NC group. Compared with the Aging + si-NC group, the tumor weight and volume of the Aging + si-CD161 group decreased (Fig. 5B-C). This shows that knocking down CD161 can inhibit tumor development.

The results of using ELISA to detect mice aging indicators: malondialdehyde (MDA), superoxide dismutase (SOD) activity, and glutathione peroxidase (GSH-Px) activity are shown in Fig. 5D. Compared with the Young group of mice, the MDA content of the aging group of mice increased significantly after the occurrence of oxidative stress, and the SOD activity and GSH-Px content significantly decreased. Oxidative stress was significantly enhanced in the aging group, and after knocking out CD161, oxidative stress was partially alleviated.

Flow cytometry was then applied to detect the expression of CD4, CD8 and CD161 in the tumor tissue, as shown in Fig. 5E. The proportions of CD161 and CD4 in the tumor tissues of mice in the Aging group were significantly higher than those in the Young group, while the proportion of CD8 was markedly higher than that in the Young group. After knocking down CD161, this trend was partially alleviated.

HE staining was used to examine tumor tissue structure and cell morphology. As can be seen in Fig. 5F, compared with the si-NC group, the tumor tissue in the Aging + si-NC group has more obvious cytoplasmic and nuclear staining, larger nucleoli, nuclear fission and nuclear overlap. Most tumor cells undergo nuclear pyknosis, and a few have nuclear disintegration, cell disintegration, and structural disappearance. Compared with si-CD161 group, Aging + si-CD161 has more obvious nuclei that are lightly stained. The cells are arranged more neatly and the nuclei overlap, nuclear fission and nuclear pyknosis were distinctly reduced, the amount of cells was obviously reduced, and nuclear lysis, cell atypia, cell disintegration and structural disappearance were reduced.

### Transcriptomic analysis

**Fig. 6:**
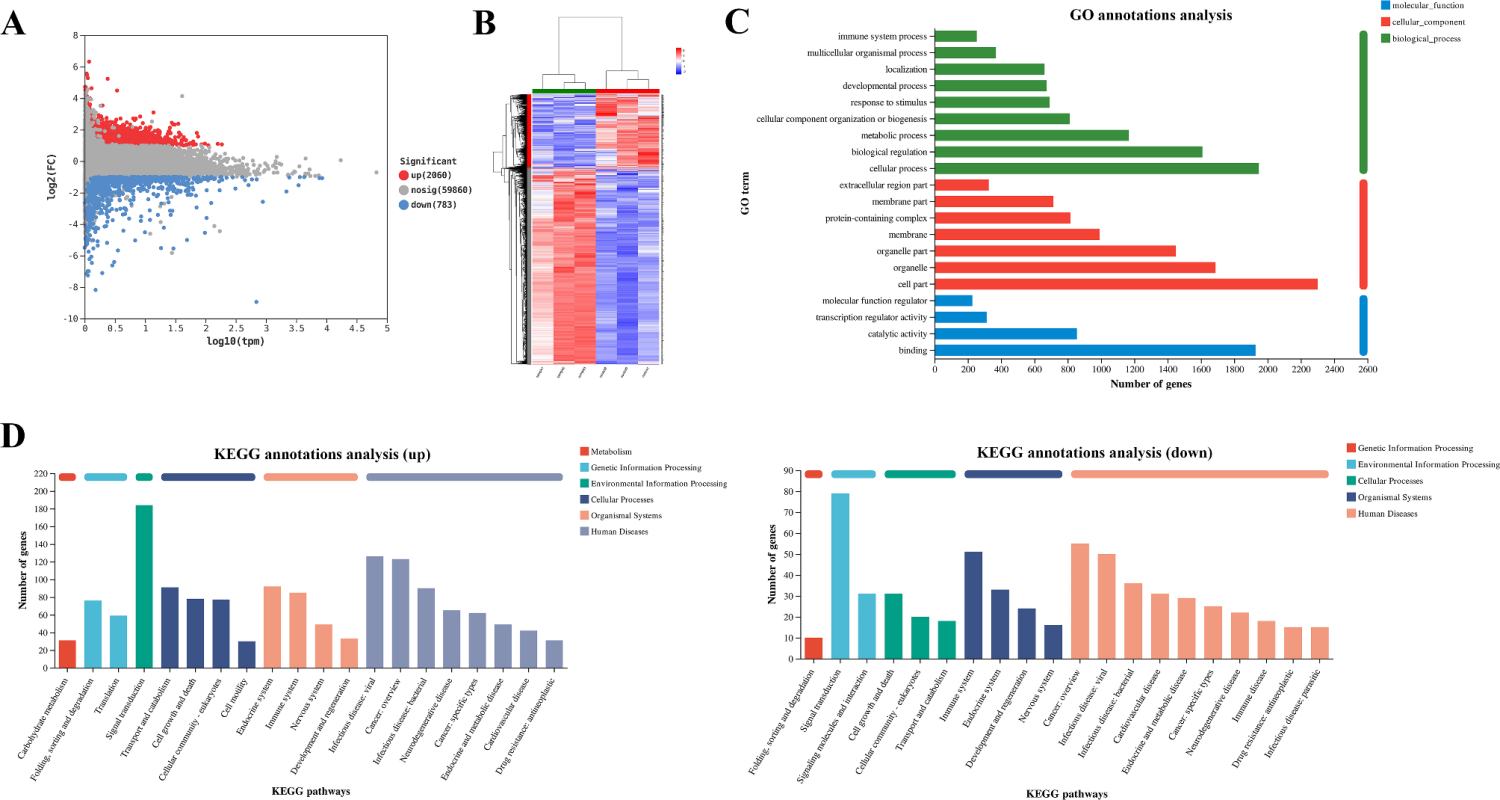
Transcriptome analysis of NKL cells before and after oxidative stress. (**A**) Differential gene expression in NKL cells before and after oxidative stress. Red, blue and gray points represent up-regulated, down-regulated and non-significant genes, respectively. (**B**) Heat map of cluster analysis of significantly differential gene expression in NKL cells before and after oxidative stress. (**C**) GO annotation analysis of DEGs in NKL cells before and after oxidative stress. (**D**) KEGG functional annotation analysis of NKL cells before and after oxidative stress. The abscissa is the number of genes annotated to this pathway. Up represents up-regulation and down represents down-regulation

**Fig. 7:**
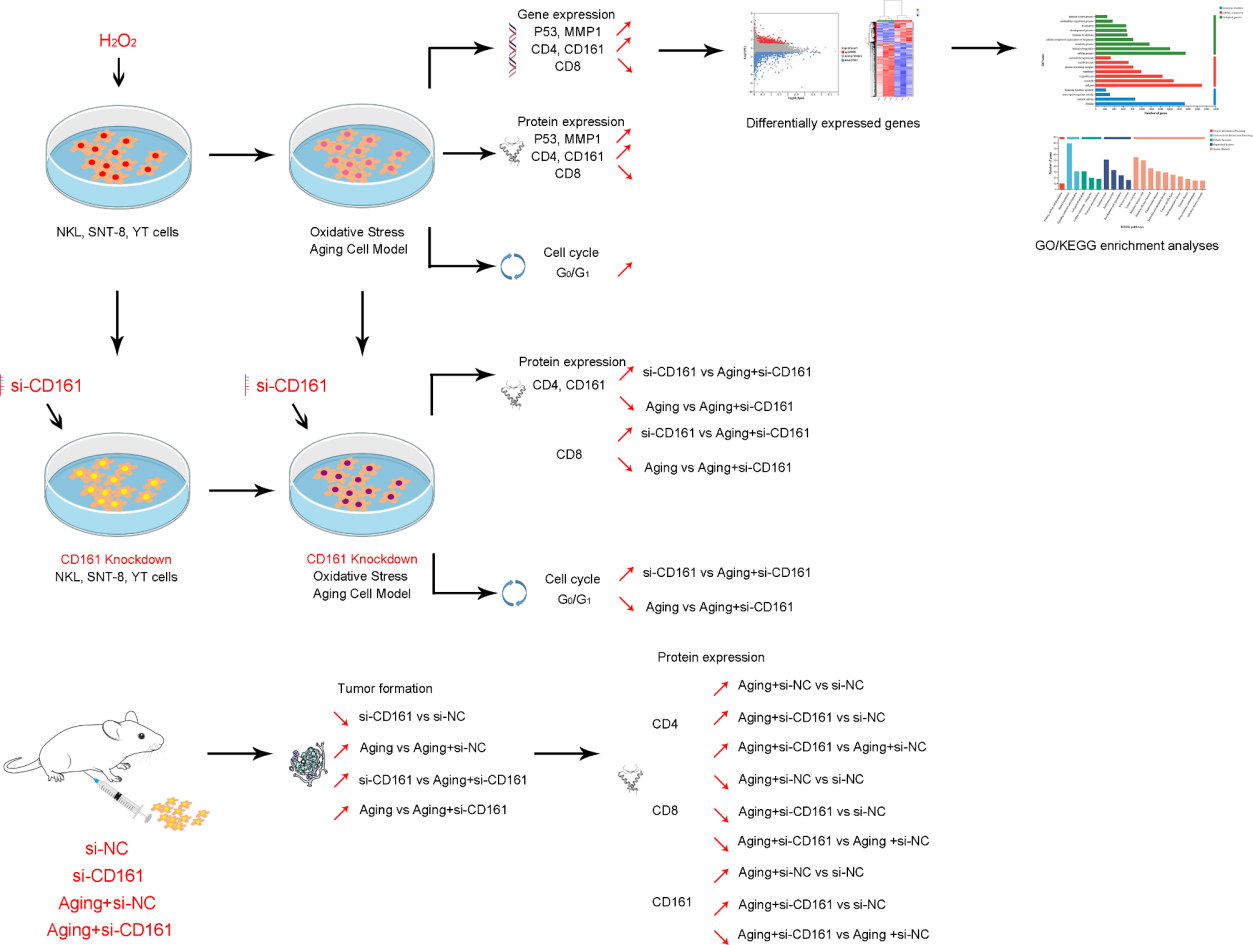
The schematic diagram of the research

Gene differential expression analysis, based on |Log2FC|>1.0 and *P* < 0.05 as the standard screening criteria, revealed a total of 2,843 differentially expressed genes (DEGs) between the Control group (Control group) and the Aging group (Sample group). Among these, there were 2,060 up-regulated genes and 783 down-regulated genes (Fig. 6A). The clustering of differential genes is shown in Fig. 6B, showing a clear separation between samples from the control and aging groups with significant color changes, indicating distinct gene expression patterns in these two groups. Although correlations among samples vary considerably, intra-group samples are closely clustered, reflecting similar gene expression profiles within each group. The results of GO annotation analysis showed that compared with the Aging group, the Control group had 2,843 DEGs classified into 52 second-level entries involving molecular function, cellular component and biological process (Fig. 6C). KEGG functional annotation analysis was performed on the DEGs in the Control group and the Aging group (Fig. 6D). The results showed that the DEGs between the Control group and the Aging group were closely related to immune function and cancer diseases. The results of Reactome annotation analysis demonstrated that DEGs are mainly concentrated in signal transduction, immune system, metabolism of proteins, disease, gene expression (transcription) and other channels (Figure S1). The results of DO annotation analysis show that DEGs are mainly concentrated in Cancer (Disease of cellular proliferation) (Figure S2). The entire experimental procedure and key findings of the current study are illustrated in Fig. 7.

## Discussion

ENKTL is a special subtype of non-Hodgkin lymphoma. It is easy to infiltrate and destroy blood vessels. It is highly invasive and has a poor prognosis. Sufficient attention should be paid to the diagnosis and treatment process. Accurate pathological examination is the main basis for diagnosis. There isn’t a consolidated and accepted protocol for handling ENKTL. Currently, treatment should be stratified by clinical stage and risk factors based on existing data (Kim et al. 2009; Breuer et al. 2012; Tse and Kwong 2017; Qi et al. 2020). Patients with stage I disease without risk factors are recommended to receive radiotherapy alone, while patients with stage I disease and stage II disease with risk factors are recommended to receive concurrent chemoradiotherapy or sequential chemoradiotherapy. For patients with stage III-IV nasal ENKTCL, L-ASP-based non-anthracycline combination chemotherapy is the first choice. When patients cannot tolerate L-ASP, pegaspargase can be used instead (Dong et al. 2016; Wang et al. 2020a, b). A portion of ENKTCL therapy involves hematopoietic stem cell transplantation, but its clinical application is limited due to high objective conditions (Yhim et al. 2015; Jeong et al. 2018; Allen and Lechowicz 2019). The clinical manifestations of ENKTCL lack specificity, and the diagnosis relies on pathological morphology and immunohistochemistry. The main treatments are radiotherapy and chemotherapy. Research on new drugs is still under development. Early diagnosis and treatment are crucial to improving the prognosis of the disease. Because there are still many problems in its diagnosis and treatment, more in-depth research by clinicians and scientific researchers is needed to guide its diagnosis and treatment.

Given that the incidence of most malignancies grows exponentially with age, age is by far the most significant risk factor for the development of cancer. Therefore, with tumor incidence rising exponentially with age, cancer continues to be the primary cause of death for both men and women aged 60 to 79 as of 2017. In the context of cancer, aging is characterized by modifications in protease depositors, mitochondrial malfunction, and epigenetic modifications (Lopez-Otin et al. 2013; Hsu 2016; Siegel et al. 2020). Inflammation is one of the signs of aging that has been connected to cancer. When inflammatory markers build up in an older adult’s blood without a microbiological infection, it’s referred to as inflammation. According to estimates, chronic, long-term inflammation accounts for 20% of cancers (Franceschi et al. 2000, 2018; Balkwill and Mantovani 2012). Cellular senescence is a steady kind of cell departure that shares biochemical similarities with terminal differentiation or cellular quiescence. Aging is a reaction to many stimuli that culminate in the activation of the DNA damage response (DDR), which in turn causes growth stop. Telomere shortening, oncogene expression, and exposure to radiation or chemotherapy are known stressors that accelerate aging (Leonardo et al. 1994; Bakkenist and Kastan 2003; Campisi and d’Adda di Fagagna 2007).

Breakthroughs in immuno-oncology research in recent years have brought about major changes in the form of tumor attack. CD161 is a protein-coding gene, and its related diseases include inflammatory bowel disease and atopic dermatitis. Related pathways include hematopoietic stem cell markers and immunomodulatory effects between lymphocytes (Beyer et al. 2000; Roda-Navarro et al. 2000; Abath Neto et al. 2014; Cheng et al. 2022); as a ligand, it interacts with CLEC2D /LLT1 binds to inhibit NK cell-mediated cytotoxicity and the secretion of interferon gamma (Oxendine and O’Connor 2021). According to reports, CD161 is an anti-tumor inhibitory receptor that T cells that infiltrate diffuse glioblastoma utilize to mediate the suppression of the CLCE2D-CD161 signaling pathway, which in turn enhances the anti-tumor immunological impact of diffuse glioma by working in concert with the anti-tumor immune response (Mathewson et al. 2021a, b). In addition, there are literatures revealing that the functional characteristics of T cells verified in glioma are also applicable to other types of tumors (melanoma, non-small cell lung cancer and colon cancer) (Inhibitory 2021; Wyrozemski and Qiao 2021; Braud et al. 2022), which suggests that CD161 can be used as a tumor infiltration function targets for immunotherapy in T cells. A recent study successfully developed a monoclonal antibody targeting CD161, demonstrating that this high-affinity antibody effectively inhibits the interaction between the inhibitory receptor CD161 and its ligand CLEC2D, thereby enhancing critical T cell functions and offering potential immunotherapeutic strategies for various hematological malignancies (Alvarez Calderon et al. 2024). These aforementioned studies underscore the pivotal role of CD161 in regulating T-cell-mediated immunotherapy across diverse malignancies, with its function predominantly linked to the mechanisms that govern the interaction between CD161 and its specific ligand CLEC2D. However, the precise T cell phenotype and the underlying mechanisms by which CD161 regulates cell cycle progression remain unclear, necessitating further investigation.

In this article, we used H_2_O_2_ to construct an aging model of oxidative stress in 3 types of cells (including NKL, SNT-8, and YT) in vitro, and we measured the senescence-related signature proteins MMP1 and p53 through Western blotting. Senescent cells usually secrete some senescence-associated secretory phenotypes (SASPs), including various inflammatory factors, growth factors, proteases and chemokines, and induce the expression of related proteins, such as MMP1 (Coppé et al. 2010; Wu et al. 2020). The increase of p53 protein level is the main cause of cell senescence and various aging phenomena. The p53 is a cell cycle regulator that activates the downstream p21 molecule, which in turn inhibits CDK2/CyclinE complex and converts phosphorylated pRb to dephosphorylated Rb under senescence. Dephosphorylated Rb protein binds to transcription factors E2F, represses transcription of genes downstream of E2F, and initiates the cellular senescence process (Herbig et al. 2004; Feng et al. 2016). In current study, the expression of MMP1 and p53 proteins were significantly increased, proving that the aging model was successfully established.

Cell cycle regulates cell division (Ettl et al. 2022), and cell cycle arrest marks the inability of cells to continue to divide, which is an important feature of cell senescence. Senescent cells are often arrested in the G0/G1 phase of the cell cycle. In the regulation of tumor cells, the arrest of tumor cells in the G0/G1 phase can inhibit the proliferation of tumor cells (Gao et al. 2021; Liu et al. 2022). The results of cell cycle detection by flow cytometry showed that the DNA replication of cells in 3 cell types aging groups was obviously blocked in the G1 phase. The findings of flow cytometry determination of the expression of the associated proteins of 3 kinds of cells revealed that compared with the Control group, the expression of CD161 and CD4 in the 3 kinds of cells aging groups were substantially higher than in the Control group, whereas the proportion of CD8 was expressively lower.

Then we constructed CD161 small interfering RNA (si-CD161), extracted the mRNA of NKL cells transfected with three siRNAs (siCD161-1, siCD161-2, siCD161-3), and finally investigated the silencing efficiency through qRT-PCR. The si-CD161-1 with the highest efficiency was used for relevant experiments. The outcomes of CCK-8 demonstrated the viability of tumor cells in the Aging group was obviously decreased after knocking down CD161, which shows that the expression of CD161 associated with aging can promote the development of tumors. The results of flow cytometry showed that cells were significantly blocked in the G1 phase. The proportions of CD161 and CD4 in the Aging group were observably increased, while the proportion of CD8 was notably decreased. BALB/c mice aging tumor model was constructed via continuous intraperitoneal injection of β-galactose for 8 weeks and subcutaneous injection of NKL cells. Knocking down CD161 can inhibit the development of tumors and partially alleviate oxidative stress.

CD4 + and CD8 + cells are different phenotypes of T lymphocytes and have an important influence on human immune response process. Various types of CD4 + T cells regulate macrophages, NK cells, and neutrophils by secreting cytokines such as interferon-γ (IFN-γ) and tumor necrosis factor α (TNF-α). Cell activity of granulocytes, etc. By measuring CD4, CD8, and CD161 in cells and mice tumor tissues, we found that the proportions of CD161 and CD4 in the Aging group were remarkably increased, whereas the expression of CD8 was markedly decreased. This may be due to the fact that CD161 inhibits the development of ENKTL tumors by regulating the cell cycle and T-cell phenotype.

Furthermore, our current study performed transcriptome sequencing of the oxidative stress-induced aging model established in our study and conducted a comprehensive analysis of the DEGs between aging cells and normal controls, aiming to elucidate the expression patterns of ENKTL-related genes upon cellular senescence. Through our analysis, a total of 2,843 DEGs were identified, with 2,060 exhibiting upregulated expression trends and 783 showing downregulated patterns. Following the enrichment analyses of these DEGs, we observed that they were significantly enriched in terms and pathways associated with immune function and cancer. Especially, Reactome annotation analysis demonstrated that they are mainly concentrated in signal transduction, immune system, metabolism of proteins, disease, and gene expression (transcription). It is currently widely accepted that the cell cycle is intricately linked to metabolism (Mukhopadhyay et al. 2015; Icard et al. 2019); consequently, these genes may influence metabolic processes and thereby impact the cell cycle. However, the mechanisms underlying CD161’s regulation of metabolism and its concurrent modulation of the cell cycle remain unclear, representing a significant avenue for our future research.

The article still has some limitations. For example, there is no intensive exploration of how CD161 affects the development of ENKTL at the pathway level. Due to the lack of bioinformatics analysis steps, senescence-related genes cannot be found by constructing a PPI network. Furthermore, the currently prevalent in vitro model of oxidative stress-induced cellular senescence, stimulated by H_2_O_2_, lacks cell aging-inducing factors that closely resemble those found in the in vivo environment, which may introduce subtle biases into the research findings. These issues require us to conduct further in-depth research and develop experimental models that more accurately reflect the conditions of the human body.

## Conclusions

In summary, our experiments studied the impact of the senescence-related gene CD161 on ENKTL, and explored the role of CD161 in ENKTL by constructing an in vitro tumor cell aging model, an in vivo BALB/c mice aging tumor model, and transcriptomic analysis. Its mode of operation is anticipated to offer fresh approaches to the management of ENKTL.

## Electronic supplementary material

Below is the link to the electronic supplementary material.

Supplementary Material 1

Supplementary Material 2

Supplementary Material 3

## Data Availability

Not applicable.
