# Supplementary material for: Expression of senescence-related CD161 promotes extranodal NK/T cell lymphoma by affecting T cell phenotype and cell cycle
Source: Mol Med. 2024 Nov 23;30:230. doi: 10.1186/s10020-024-00969-7 (PMC11585959; doi:10.1186/s10020-024-00969-7)
Supplement: Supplementary file 3 — Supplementary Material 3 [file 10020_2024_969_MOESM3_ESM.docx]

**Author Response to Reviewers**

Dear Editors and Reviewers:

We sincerely appreciate your letter and the reviewers' insightful comments regarding our manuscript entitled “Expression of Senescence-related CD161 Promotes Extranodal NK/T Cell Lymphoma by Affecting T Cell Phenotype and Cell Cycle”. Those comments provided are all valuable and greatly assist in revising and enhancing our paper, as well as offering significant guidance for our research. We have studied the comments carefully and have made corrections which were marked in track changes mode. The main corrections in the paper and the responses to the reviewers’ comments were listed as follows. We would like to re-submit this revised manuscript to your journal and hope that the correction will meet with approval.

Thank you once again for considering the publication of our manuscript. If you have any additional questions, please contact us without hesitation.

Best regards,

Chaohe Zhang

**Responds to Reviewer 1’s comments**

Reviewer 1: The manuscript entitled "Expression of Senescence-related CD161 Promotes Extranodal NK/T Cell Lymphoma by Affecting T Cell Phenotype and Cell Cycle" explores the role of CD161 in extranodal NK/T cell lymphoma (ENKTL). The authors have conducted comprehensive experiments and provided substantial supporting data. However, there are several areas where the manuscript could be improved for clarity and impact.

The abstract needs refinement for better clarity. Ensure that all abbreviations, such as NK, SNT, and others, are fully defined upon first use. Additionally, the rationale for focusing on CD161 should be more robust, providing a clearer context based on previous findings related to CD161 in immune regulation and cancer. Explicitly stating the hypothesis at the end of the introduction would better guide the reader.

**Response:** We appreciate your valuable suggestion. Following your suggestion, we have refined the abstract of the manuscript and provided definitions for any abbreviations introduced for the first time in the abstract. However, the abbreviations you referenced, including NKL, SNT-8, and YT, are the names of cell lines without accompanying full names. Furthermore, we have incorporated a concise overview of the role of CD161 in cancer within the introduction of the revised manuscript and concluded it with our proposed hypotheses for this study. We anticipate that these revisions will meet your approval and engage the readers' interest.

The Materials and Methods section should be re-written to describe the experimental procedures in precise scientific language, avoiding instructional tone. For example, instead of saying, "Take logarithmic growth phase cells and inoculate them into 1 mL or 2 mL at 1 × 10^6 cells/mL in a 24-well plate 6-well plate," it should detail the exact procedures performed by the authors. Additionally, the purpose and effects of H2O2 treatment on the cells need clearer explanation. It is introduced in the transcriptomic analysis section but lacks sufficient detail in the methodology section.

**Response:** Thank you for your pivotal suggestion. We have rewritten the Materials and Methods section using precise scientific language to describe the experimental and analytical procedures of our research. Additionally, the methodology employed for treating the cell with H_2_O_2_ has been further elaborated in the Materials and Methods section, thereby enhancing readers' understanding of the cell models utilized in our study.

In the Results section, the choice of aging markers, specifically the use of p53 and MMP1, should be justified. Consider incorporating more established senescence markers such as Senescence-Associated β-Galactosidase, CDKN2A (p16^INK4a), or HMGB1. Moreover, the presentation of the results should be in clearer scientific terms. For instance, phrases like "CD161 small interfering RNA (si-CD161) was constructed, the mRNA from NKL cells transfected with three siRNAs (siCD161-1, siCD161-2, siCD161-3) were extracted, and the silencing efficiency was determined via qRT-PCR" should be simplified for better readability.

**Response:** We would like to express our gratitude for your valuable suggestion. Following a thorough review, we have implemented comprehensive revisions to the scientifically flawed statements we identified. However, we are currently unable to enhance our research with experiments aimed at detecting additional aging biomarkers due to funding constraints. In light of this, we sincerely extend our apologies to you. We will take your advice into consideration in our future work and design and execute more compelling experimental investigations in our future research.

The discussion section lacks depth in explaining the mechanisms. Expand on how CD161 might influence T cell phenotype and cell cycle regulation. Additionally, a more extensive comparison with existing literature is needed to contextualize the findings. Discuss potential biases, limitations of the in vitro and in vivo models, and any technical constraints. Suggest specific future experiments, such as pathway-specific analyses or using humanized mouse models, to address these limitations.

**Response:** Thank you for your crucial suggestion. We have made a comprehensive revision of the discussion section. We have expanded our discussion on the mechanisms by which CD161 regulates T cells and addressed several limitations of the study, including those associated with the cell model.

Emphasize the potential clinical relevance of the findings, particularly how targeting CD161 could be developed into a therapeutic strategy for ENKTL. Additionally, the manuscript should address several critical questions: How does the expression of CD161 in ENKTL compare to other types of lymphomas or cancers? Are there specific ligands or receptors interacting with CD161 that could be explored further? Can more detailed insights into the signaling pathways involved be provided, identifying the upstream regulators and downstream targets of CD161? Would additional functional assays, such as T cell cytotoxicity or cytokine production assays, provide more insights into the role of CD161? The in vivo experiments show that knocking down CD161 inhibits tumor growth. How do these findings translate to potential therapeutic applications in humans? Are there any ongoing clinical trials targeting CD161 or related pathways in ENKTL or other cancers?

**Response:** We appreciate your valuable questions which can enhance our manuscript and our research horizon. However, we are currently limited to incorporating comparisons of our findings with those from other studies within the discussion. Regarding the questions you raised about clinical relevance and subsequent mechanism studies, we recognize the need for further investigations in the future to expand upon this research. Regrettably, we are unable to address all of your inquiries within this article. We would like to express our sincere apology for this and appreciate your understanding.

**Responds to Reviewer 2’s comment**

Reviewer 2: Article by Dr. Zhang and group elaborating the role of CD161 Affecting T Cell Phenotype and Cell Cycle. This is a very well written article, follows through hypothesis of work; very well planned experiments with clear results. Though few things must be addressed before it is ready for acceptance. They are as follows:

1. In this paper, Fig S1, it has been shown that, from Reactome annotation analysis demonstrated that DEGs are mainly concentrated in signal transduction, immune system, metabolism of proteins and overall metabolism along with cell cycle. Cell cycle is significantly interconnected with metabolism. It has been well demonstrated in PMID: 26682255 and PMID: 30655165. Authors must add their their perspective on this topic, related to the finding of this research article. This is a provocative topic for future studies, how CD161 would be regulating metabolism while it modifies cell cycle regulation. Authors should add few lines on this in the discussion part, by adding the mentioned relevant referred works.

**Response:** Thank you for your pivotal suggestion. In the Discussion section of the revised manuscript, We have supplemented our discussion regarding the implications of Reactome annotation analysis results in relation to the cell cycle. Furthermore, we have also delineated potential avenues for our future research.

2. Authors must add a model at the last figure, depicting the major findings from this work and showing how this fits in the known facts in the field.

**Response:** Thank you for your detailed suggestion. We have presented a schematic diagram of the entire research, which is illustrated as Figure 7. We believe that the diagram we have provided will offer the author a more lucid perspective on their research.

3. All the texts, in any figures must be in bigger size and with proper resolution, so that it is readable. This must be rectified.

**Response:** We would like to express our gratitude for your pivotal suggestion. We have already re-uploaded all figures of our manuscript in an enlarged format and with appropriate resolution to enhance the readability of our work.

Special thanks to you for your good comments.

We appreciate for reviewers’ warm work earnestly, and hope that the correction will meet with approval.

Once again, thank you very much for your comments and suggestions.

Yours Sincerely

Chaohe Zhang
